# Impact of Non-Valvular Non-Coronary Concomitant Procedures on Outcomes of Surgical Aortic Valve Replacement in Intermediate Risk Patients

**DOI:** 10.3390/jcm10235592

**Published:** 2021-11-28

**Authors:** Fanar Mourad, Ali Haddad, Janine Nowak, Mohamed Elbarraki, Yacine Elhmidi, Marinela Jasarevic, Philipp Marx, Ender Demircioglu, Daniel Wendt, Matthias Thielmann, Bastian Schmack, Arjang Ruhparwar, Sharaf-Eldin Shehada

**Affiliations:** 1 Department of Thoracic and Cardiovascular Surgery, West German Heart and Vascular Center, University Hospital Essen, 45147 Essen, Germany; fanar.mourad@uk-essen.de (F.M.); janinenowak@yahoo.de (J.N.); mo.elbarraki@gmail.com (M.E.); marinela.jasarevic@uk-essen.de (M.J.); Philipp.marx@uk-essen.de (P.M.); ender.demircioglu@uk-essen.de (E.D.); daniel.wendt@uk-essen.de (D.W.); matthias.thielmann@uk-essen.de (M.T.); bastian.schmack@uk-essen.de (B.S.); Arjang.Ruhparwar@uk-essen.de (A.R.); 2 Department of Anaesthesiology and Intensive Care Medicine, University Hospital Essen, 45147 Essen, Germany; ali.haddad@uk-essen.de; 3 Department of Cardiac Surgery, Ludwigshafen Heart Centre, 67063 Ludwigshafen, Germany; elhmidiy@klilu.de

**Keywords:** surgical aortic valve replacement, intermediate risk patients, isolated and combined aortic valve replacement

## Abstract

Introduction: advanced age and concomitant procedures could increase the risk of perioperative complications during surgical aortic valve replacement (SAVR). We aimed to evaluate results of elderly patients undergoing SAVR and evaluate the impact of concomitant non-valvular, non-coronary procedures on the outcomes. Methods: A retrospective single-centre study, evaluating 464 elderly patients (mean age = 75.6 ± 4 years) undergoing either isolated-SAVR (I-SAVR = 211) or combined-SAVR (C-SAVR = 253) between 01/2007 and 12/2017. Combined-SAVR involved non-valvular, non-coronary procedures. Study endpoints are postoperative results concerning the VARC-II criteria, valve dysfunction, long-term freedom from redo-AVR and survival. Results: males were 52.8%. Patients had an intermediate risk profile (mean EuroSCORE-II (%) 5.2 ± 5). Postoperative results reported no significant differences in incidence of re-exploration for bleeding (6.6% vs. 6.7%, p = 1.0), stroke (0.9% vs. 0.4%, p = 0.59), dialysis (6.2% vs. 9.5%, p = 0.23) and pacemaker implantation (3.3% vs. 2.8%, p = 0.79) between I-SAVR and C-SAVR groups. Thirty-day (2.4% vs. 7.1% p = 0.03), one-year (5.7% vs. 13.8%, p = 0.003) and overall mortality (24.6% vs. 37.5%, p = 0.002) were lower in the isolated-SAVR group. Re-AVR was indicated in 1.7% of patients due to endocarditis. Conclusions: SAVR in elderly patients offers good outcomes with increased life quality and rare re-operation for structural valvular deterioration. Mortality rates were significantly higher when SAVR was combined with another “non-valvular, non-coronary” procedure.

## 1. Introduction

Age distribution of cardiac surgery patients in Germany shows an increased shift toward an elderly population. In 2019, the German Heart Surgery Report reported that more than 53% of cardiac procedures were performed in patients older than 70 years during the last ten years [1]. It registered a total of 174,902 cardiac procedures, of these 18.75% (32,810) were aortic valve procedures; most of them (59.2%) were either isolated or combined surgical aortic valve replacement (SAVR) [1]. SAVR can be performed either conventional or minimally invasively via partial sternotomy or right lateral mini-thoracotomy [2,3]. Recently, trans-catheter aortic valve implantation (TAVI) has been more frequently adopted in isolated aortic valve procedure in high-risk patients, then in intermediate risk and now in low risk patients [4,5]. In Germany, the number of TAVI procedures increased from 2198 in 2009 to 13,279 in 2018, which represents 57.5% of the isolated aortic valve procedures [1]. Different strategies, implantation modifications and different outcomes and complications have been widely discussed for the TAVI procedures [6,7,8]. However, combined non-valvular and non-coronary pathologies, including ascending aorta aneurysm, septum hypertrophy, small annuli, atrial fibrillation (AF) and patent foramen ovale (PFO), are factors leading to SAVR procedure. We therefore aimed to evaluate the additional risk of morbidity and mortality of those combined pathologies and procedures on intermediate risk patients undergoing SAVR.

## 2. Patients and Methods

### 2.1. Study Design and Patients

This is a retrospective single-centre study that evaluates 464 consecutive elderly (≥70 years) patients undergoing SAVR between 01/2007 and 12/2017. They undergo either isolated or combined SAVR with non-valvular non-coronary procedures: Ascending aorta repair/replacement, aortic root enlargement, sub-valvular myectomy or decalcification, PFO or left atrial appendage closure, ablation and intra cardiac tumour resection. Patients with combined SAVR with composite conduit (i.e., David, Yacoub or Bentall), aortic arch surgery, mitral or tricuspid valve procedures or coronary artery bypass grafting and redo procedures were excluded. The study obtained a review board approval according to the University Hospital Ethics Committee (Ref# 18-8421-BO).

### 2.2. Data Collection and Follow-Up

Patients’ preoperative, operative and postoperative data were recorded in our institutional database. A retrospective data extraction and evaluation was performed. A follow-up was performed by reviewing medical records and communication with civil office as well as an active personal or phone-call interview using a standardized questionnaire that was established in reference to the EuroQol-questionnaire to evaluate patients’ general and clinical status. The follow-up was continued until an endpoint of death or completion of the study through September 2020. The follow-up for survival was 100% completed; however, 23 survivals did not fill out the questionnaire, resulting in 95% completion for the clinical follow-up.

### 2.3. Study Endpoints

Primary endpoints were postoperative morbidities in reference to the Valve Academic Research Consortium II initiative (VARC II) criteria, which included 30-day mortality, incidence of stroke, myocardial infarction, re-exploring for bleeding, acute kidney insufficiency, new haemodialysis, pacemaker implantation and new onset of arterial fibrillation. Secondary endpoints were freedom of re-aortic valve replacement (AVR) or valve dysfunction and overall long-term survival.

### 2.4. Statistics

A descriptive statistical analysis was performed using the SPSS-software (version 22.0. IBM Corp., Armonk, NY, USA). Continuous data were expressed as mean ± standard deviation (SD) or median with interquartile ranges (IQRs) (25–75th percentiles) and compared between groups using the unpaired Student’s *t*-test or Mann–Whitney U test when appropriate. Categorical data were expressed as frequencies and percentages and compared between groups using Chi-Square (χ^2^) test or Fisher’s exact test. Reported *p*-values are two-sided and a value of *p* < 0.05 was considered statistically significant. Additionally, Kaplan–Meier curves were generated using the R software to estimate freedom from cardiac or all-cause mortality in both groups; log-rank test was used to evaluate differences between both groups.

## 3. Results

### 3.1. Patient Population

A total of 464 consecutive elderly patients undergo either isolated-SAVR (I-SAVR) in 211 (45.5%) patients or combined-SAVR (C-SAVR) with non-valvular, non-coronary procedure in 253 (54.5%) patients. Detailed baseline characteristics are summarized in Table 1. Mean age was 75.6 ± 4 years and the cohort included more males (52.8%), where 6% of the patients presented for an urgent/salvage procedure. Risk scores in both groups reported intermediate risk with significant higher risk scores in the C-SAVR group: Logistic EuroSCORE I (15.2 ± 12.3 vs. 9.6 ± 7, *p* < 0.0001), EuroSCORE II (6.7 ± 6.2 vs. 2.9 ± 2.4, *p* < 0.0001). Patients in the I-SAVR presented with advanced NYHA classifications NYHA III-IV (60.7% vs. 43.5%, *p* < 0.0001) compared to C-SAVR patients. Table 2 contains preoperative echocardiographic data. Most of the patients presented with either aortic valve stenosis (50.4%) or concomitant stenosis with regurgitation (42.5%). Morphologically 80.4% of the patients have tricuspid aortic valve with a mean orifice area of 0.93 ± 0.3 cm^2^ and a mean gradient of 50.6 ± 23.3 mmHg. Impaired left ventricular function was reported in 94 (20.2%) patients.

### 3.2. Operative Outcomes

Table 3 summarizes the operative outcomes. A minimally invasive procedure via partial sternotomy was performed in 26.5% of patients. Most patients received biological prosthesis (97.6%) with a mean size of 23 ± 2 mm. C-SAVR patients have longer cross clamp time and CPB times (69.9 ± 23.4 vs. 60.7 ± 17.3 min, *p* < 0.0001 and 101.9 ± 37.9 vs. 88.9 ± 25.1 min, *p* < 0.0001) than isolated SAVR. The same group required more intraoperative foreign blood transfusion (587.8 ± 493.5 vs. 449.9 ± 435.8 mL, *p* < 0.0001). C-SAVR patients underwent one or more concomitant procedures, which were mainly sub-valvular myectomy and/or decalcification (70%), ascending aorta repair/replacement (30%), aortic root enlargement (8.7%), closure of a patent foramen ovale (9.1%), left atrial appendage occlusion or rhythm procedure (13.4%) or resection of an atrial tumour (1.4%).

### 3.3. Postoperative Outcomes

Postoperative results show no difference between I-SAVR and C-SAVR patients in regard to myocardial infarction (0.5% vs. 0%, *p* = 0.46), revision for bleeding (6.6% vs. 6.7%, *p* = 1.0), need for temporary dialysis (6.2% vs. 9.5%, *p* = 0.23), pacemaker implantation (3.3% vs. 2.8%, *p* = 0.79) or new onset of atrial fibrillation (33.6% vs. 27.6%, *p* = 0.19). Combined-SAVR has significantly more 30-day mortality (7.1% vs. 2.4%, *p* = 0.03) but similar cardiac-related mortality (2.8% vs. 1.9%, *p* = 0.76) as reported in Table 4.

### 3.4. Late and Follow-Up Outcomes

**Figure 1:**
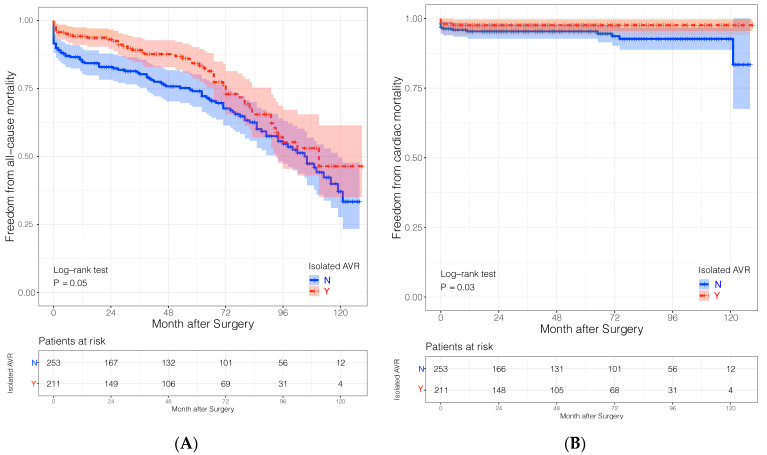
Kaplan–Meier curves showing: (**A**) Freedom from all-cause mortality in both subgroups. (**B**) Freedom from cardiac mortality in both subgroups.

Survival follow-up was 100% completed and is reported in Table 4, showing higher incidence of cumulative one-year (13.8% vs. 5.7%, *p* = 0.003), five-year (13.8% vs. 5.7%, *p* = 0.003) and overall (37.5% vs. 24.6%, *p* = 0.002) mortalities in the combined-SAVR group, as also illustrated with Kaplan–Meier curves (Figure 1A,B), and showing significant lower cardiac mortality (log-rank = 0.03), but slightly non-significant all-cause (log-rank = 0.05) mortality in the I-SAVR group. The random survival forest revealed that concomitant SAVR was a significant factor associated with all-cause mortalities (*p* = 0.002) and cardiac mortalities (*p* = 0.01). More details can be found in the Supplementary Table S1. Table 5 summarizes clinical outcomes of the survivals: No differences were recorded between both groups regarding incidence of structural prosthesis deterioration, indicating reoperation (1.4% vs. 2%, *p* = 1.0) or prosthesis dysfunction not indicating re-surgery (3.5% vs. 4.6%, *p* = 0.88). The indication for re-SAVR was severe destructive endocarditis in all patients. Furthermore, no differences were observed in the incidence of stroke (4.9% vs. 5.9%, *p* = 0.8), myocardial infarction (1.4% vs. 0%, *p* = 0.88), or coronary revascularization (1.4% vs. 2.6%, *p* = 0.69) between both groups, respectively. Most of the survivals were physically independent (73.5%) and presented with NYHA I-II classification (74.8%).

## 4. Discussion

The main findings of this study were: 1. There were no significant differences between the two groups in regard to VARC II criteria except for all-cause early mortality. 2. Patients undergoing SAVR with combined procedures have a two- to threefold higher postoperative mortality than those without.

Different outcomes of SAVR in elderly patients have been reported based on patients’ clinical status, comorbidities, procedural time, as well as concomitant procedures. In this study, 464 consecutive elderly patients (≥70 years) who underwent SAVR were evaluated; a 30-day mortality was reported in 4.9% of patients, significantly lower in the isolated-SAVR group (2.4% vs. 7.1%, *p* = 0.03). To be precise, investigation of the deaths and cardiac mortality was reported in 2.4% of patients. Moreover, most (12/23) of the patients who died had higher risk scores (log. EuroSCORE 24.3 ± 4.2%); this decreased with time after the increased adoption of TAVI procedure in high-risk patients; similarly, in this study, early mortality decreased to 3.5% (9/255) after 2010. Thus, when looking at patients with lower-risk scores in this cohort, 30-day mortality was reported in only 0.7% (1/149) of patients, which in turn is comparable to the recently published TAVI in low risk-data [4,5]. Early mortality was reported in 2.4% of patients in isolated-SAVR, which was slightly lower than the unadjusted mortality rate of isolated-SAVR in the German annual report, which varies between 2.6% and 2.9% within the last 10 years [1], and the crude 3.1% (95% CI, 2.6–3.7%) mortality rate of SAVR without concomitant-CABG for elderly patients in the united states between 1999 and 2011 [9].

Long-term outcomes after TAVI are still under investigation. Some investigators reported comparable five-year mortality between TAVI and SAVR in intermediate and high-risk patients [10,11]. In the recently published meta-analysis evaluating different randomized trials from Barili et al. survival advantage was observed in patients undergoing TAVI in the first year after the procedure; this however was changed later on, where at 40 months the survival rates would favour SAVR patients; this was attributed to other factors involving perioperative pacemaker implantation, significant perivalvular leaks and durability of the TAVI-prosthesis [12]. In our study, the estimated 10-year survival reaches around 40%, which is similar to early reported data from other investigators [13,14].

The impact of concomitant non-valvular, non-coronary procedures on outcome after SAVR is not widely discussed, such as ascending aorta repair or replacement, aortic root enlargement, sub-valvular myectomy or decalcification, which sometimes reach down to the anterior mitral leaflet. In fact, all these concomitant procedures subsequently increase of intraoperative complications or postoperative bleeding with immense prolonged postoperative course and hence the risk of mortality. Thus, the question is whether there is any benefit to performsuch concomitant procedures during SAVR. Such pathologies are not addressed during treatment within TAVI procedures.

Earlier studies addressing this reported that SAVR could have a protective role in the progression of ascending aorta dilatation as well as hypertrophy of the left ventricular septum [15,16]. Others reported no increase in mortality rate after selected concomitant procedures including root enlargement, sub-valvular myectomy and ablation procedures [17,18,19]. In fact, our study demonstrates significant lower early (2.4% vs. 7.1%, *p* = 0.03) and late mortalities (24.6% vs. 37.5%, *p* = 0.002) in the isolated SAVR group than the combined-SAVR with non- valvular, non-coronary procedure. Of note, most of the patients who died (11 out of 18 for the 30-day and 51 out of 95 for the late mortalities) in the second group underwent concomitant sub-valvular myectomy. In our study, about 45.5% (115/253) of patients in the combined group presented for surgery in advanced status of left ventricular hypertrophy, which has already been reported as a negative predictor of early mortality in SAVR [20]. Additionally, 19% (48/253) of the patients had atrial fibrillation, which also would be associated with a more than two-fold increased risk of cardiac and all-cause mortality after TAVI and considered as an independent predictor of late adverse cardiac and cerebrovascular events after SAVR [21]. Earlier aortic valve intervention for patients with moderate to severe aortic stenosis with increasing left ventricle mass even if they are asymptomatic has been reported to have better early and late survival [22]. Therefore, frequent clinical and echocardiographic examination to identify early and treat aortic stenosis in those patients, besides optimizing therapy for the risk factor, is essential to improve outcomes.

This reflects the importance of the right timing of aortic valve stenosis intervention rather than judging the procedure (SAVR or TAVI) itself. Small aortic annuli is a competitive finding facing both SAVR and TAVI, which might easily result in patient prosthesis mismatch (PPM), resulting in decreased long-term survival and increased rehospitalisation due to heart failure or for re-SAVR [23]. Concomitant aortic root enlargement is considered as an effective method to avoid PPM in small aortic annuli, with no increase in the early mortality, and allows better long-term outcomes [17]. Advanced technology of valve production in the current era provides another opportunity for those patients; it aims to avoid PPM similar to aortic root enlargement, involving the use of supra annular stented prostheses [24,25] or sutureless prostheses, which have no suturing ring and accordingly increased effective valvular orifice area [26]. These alternatives could facilitate procedure and decreases the rate of root enlargement-associated complications. Of note, transcatheter prostheses could present a significantly better effective orifice area and maybe should be kept in mind as a good alternative in challenging obese patients with small aortic annuli.

Generally, the main purpose of ascending aorta repair or replacement is to prevent acute aortic events (dissection, rupture, pseudo-aneurysm). The guidelines depend in this regard mostly on the diameter of the aorta to identify the indication for surgery; the current ones indicate concomitant aortic repair in patients undergoing aortic valve replacement when the ascending aortic diameter ≥ 45 mm [27]. Several studies showed low expansion rate and acute aortic events and rare or no reoperation on a dilated ascending aorta after isolated SAVR in cases of tricuspid aortic valves within mid- to long-term follow-up [15]. This differs in cases of bicuspid aortic valve (BAV), Yasuda et al. reported further dilation of ascending aorta after isolated SAVR and attributed that to the fragility of the aortic wall rather than the hemodynamic factors [28]; other investigators reported stable diameter of dilated aorta in stenotic BAV patients for at least 10 year after isolated SAVR [29]. Recently, encouraging early and mid-term outcomes have been reported for patients with aneurysmatic ascending aorta undergoing only TAVI [30]. In this study, 30% (76/253) of the combined-group patients underwent ascending aorta repair or replacement, where 30-day, one-year and late mortalities were observed in 3/76 (3.9%), 10/76 (13.2%) and 20/76 (26.3%) of the patients, respectively. Therefore, after considering the guidelines’ recommendations, the indication for concomitant ascending aorta procedure in elderly patients during SAVR could be limited to a few categories, e.g., patients with connective tissue disorders or those with familial aortic aneurysms and in selected BAV phenotypes.

In the current study, redo-AVR was reported in 5 (1.1%) patients due to prosthesis endocarditis and not due to structural valve deterioration (SVD). Interestingly, during clinical and echocardiographic follow-up a few patients had subclinical bio-prosthesis changes without the need for re-AVR, which are presented as a mild increase in the transvalvular gradient in 3/294 (1%), mild aortic insufficiency in 6/294 (2%), as well as prosthesis endocarditis in 3/294 (1%) patients, which were treated conservatively with antibiotics. A possible explanation of this low rate of SVD could be the used prosthesis where Carpentier–Edwards Perimount prosthesis (Edwards Lifesciences, Irvina, CA, USA) and St. Jude Trifecta prosthesis (St. Jude Medical, St. Paul, MN, USA) was implanted in 67.2% (312/464) and in 23.9% (111/464) of patients, respectively. Those prostheses have reported excellent durability and low SVD rates, especially in elderly patients [31,32].

Finally, a long-term clinical follow-up at 88.8 ± 39.4 months shows significant improvement of life quality and absence of symptoms after surgery for those elderly patients. Most patients (73.5% (216/294)) were independent of help and 74.8% (220/294) presented with NYHA I-II, with low incidence rates of late stroke (16/294, 5.4%), myocardial infarction (2/294, 0.7%) and pacemaker implantation (15/294, 5.1%), which is comparable with results after TAVI in low-risk patients [4,5]. This quality of life benefit is in accordance with the early reported data of a large meta-analysis reviewing postoperative health-related quality of life (HRQOL) after SAVR in elderly that suggests evaluating patients for surgery based on their comorbidities rather than their age [33].

## 5. Conclusions

SAVR in intermediate risk patients was associated with acceptable early and long-term outcome with increased life quality and re-operation for structural prosthesis deterioration was rare. Interestingly, some non-valvular, non-coronary concomitant procedures seem to have a negative impact on results. This could be due to the advanced underlying pathologies and delayed surgical timing. Thus, frequent echocardiographic and clinical observation of those patients and discussing of their findings in heart team to choose optimal time, treatment strategy and indication of concomitant procedure is mandatory to improve outcomes.

## 6. Study Limitation

Our study was performed at a single centre with a relatively small cohort; however, it presents long-term outcomes for elderly patients who underwent either isolated or combined non-valvular, non-coronary SAVR, which so far not addressed in the literature. The heterogeneity between both groups in regards to preoperative data could affect results; a matching analysis was not performed due to the small sample size in each group, even though, these data present a part of our daily practice and a kind of real-world outcome. The nature of the study being a retrospective one, and finally the absence of a comparison group undergoing TAVI are limitations of this study.

## Figures and Tables

**Table 1:** Baseline characteristics.

Variable	Total *n* = 464	Isolated–SAVR *n* = 211	Combined-SAVR *n* = 253	*p*-Value
Demographics				
Age, years	75.6 ± 4	75.2 ± 3.6	75.9 ± 4.3	0.06
Gender, male	245 (52.8)	123 (58.3)	122 (48.2)	0.03
BMI *, kg/m^2^	27.8 ± 5.1	28.2 ± 4.8	27.5 ± 5.3	0.14
Risk factors & comorbidities				
Peripheral vascular disease	34 (7.3)	17 (8.1)	17 (6.7)	0.59
Hypertension	397 (85.6)	176 (83.4)	221 (87.3)	0.24
COPD *	89 (19.2)	48 (22.7)	41 (16.2)	0.08
Diabetes mellitus	115 (24.8)	53 (25.1)	62 (24.5)	0.91
Pulmonary hypertension	78 (16.8)	39 (18.5)	39 (15.4)	0.39
Hyperlipidaemia	229 (49.3)	94 (44.5)	135 (53.4)	0.06
Prior dialysis	7 (1.5)	4 (1.9)	3 (1.2)	0.71
Prior myocardial infarction	18 (3.9)	7 (3.3)	11 (4.3)	0.64
Prior cerebrovascular accident	36 (7.7)	11 (5.2)	25 (9.9)	0.08
Prior pacemaker implantation	25 (5.4)	10 (4.7)	15 (5.9)	0.68
NYHA * III-IV	238 (51.3)	128 (60.7)	110 (43.5)	<0.0001
Urgent/Emergent indication	28 (6.0)	15 (7.1)	13 (5.1)	0.31
Risk-scores				
Logistic EuroSCORE I	12.6 ± 10.6	9.6 ± 7	15.2 ± 12.3	<0.0001
EuroSCORE II	5.2 ± 5	2.9 ± 2.4	6.7 ± 6.2	<0.0001
STS-PROM	2.3 ± 1.7	2.3 ± 1.6	2.4 ± 1.8	0.411

Data are presented as mean ± SD or number (%). * BMI = Body mass index; * COPD = Chronic obstructive pulmonary disease; * NYHA = New York Heart Association.

**Table 2:** Preoperative echocardiographic data.

Variable	Total *n* = 464	Isolated–SAVR *n* = 211	Combined-SAVR *n* = 253	*p*-Value
Aortic valve Pathology				
Isolated stenosis	234 (50.4)	109 (51.7)	125 (49.4)	0.64
Isolated regurgitation	25 (5.4)	6 (2.8)	19 (7.5)	0.04
Combined stenosis & regurgitation	197 (42.5)	93 (44.1)	104 (41.1)	0.57
Aortic valve endocarditis	8 (1.7)	3 (1.4)	5 (2.0)	0.73
Aortic valve morphology				
Unicuspid	2 (0.4)	1 (0.5)	1 (0.4)	1.0
Bicuspid	89 (19.2)	36 (17.1)	53 (20.9)	0.34
Tricuspid	373 (80.4)	174 (82.4)	199 (78.7)	0.35
Impaired Left ventricle ejection friction				
EF < 30%	10 (2.1)	4 (1.9)	6 (2.4)	0.76
EF 30–50%	84 (18.1)	43 (20.4)	41 (16.2)	0.28
Aortic valve orifice area (cm^2^)	0.93 ± 0.3	0.91 ± 0.3	0.96 ± 0.3	0.04
Mean gradient, mmHg	50.6 ± 23.3	48.5 ± 21.2	54 ± 26.1	0.19

Data are presented as Mean ± SD or number (%). EF = ejection fraction.

**Table 3:** Operative outcomes.

Variable	Total *n* = 464	Isolated–SAVR *n* = 211	Combined-SAVR *n* = 253	*p*-Value
Biological prosthesis	453 (97.6)	209 (99.1)	244 (96.4)	0.07
Prosthesis size, mm	23 ± 2	23 ± 2	23 ± 2	0.88
Aortic cross clamp time, min	66 ± 21	61 ± 17	70 ± 23	<0.0001
Intraoperative Blood transfusion, ml	525 ± 490	450 ± 436	588 ± 494	<0.0001
Concomitant Procedures				
Ascending aorta repair/replacement	76	-	76 (30)	-
Aortic root enlargement	22	-	22 (8.7)	-
Sub-valvular myectomy/decalcification	177	-	177 (70)	
* PFO closure	23	-	23 (9.1)	-
* LAA occlusion ± Ablation	34	-	34 (13.4)	-
Atrial tumours resection	4	-	4 (1.6)	-

Data presented as mean ± SD or number (%). * PFO = Patent Foramen Ovale; * LAA = Left atrial appendage.

**Table 4:** Postoperative and survival outcomes.

Variable	Total *n* = 464	Isolated–SAVR *n* = 211	Combined-SAVR *n* = 253	*p*-Value
**Early outcomes**				
Ventilation time, hours	10 (7–18)	10 (6–18)	10 (7–17)	0.21
Intensive-care stay, hours	25 (21–70)	25 (21–50)	28 (21–91)	0.94
Blood transfusion, ml	600 (0–600)	300 (0–600)	600 (0–600)	0.07
Re-exploration for bleeding	31 (6.7)	14 (6.6)	17 (6.7)	1.0
Deep wound infection	2 (0.4)	2 (0.8)	0	0.21
Low cardiac output syndrome	14 (3.0)	4 (1.9)	10 (3.9)	0.28
Myocardial infarction	1 (0.2)	1 (0.5)	0	0.46
Temporary dialysis	37 (8.0)	13 (6.2)	24 (9.5)	0.23
Re-Intubation	25 (5.4)	12 (5.7)	13 (5.1)	0.84
Stroke	3 (0.6)	2 (0.9)	1 (0.4)	0.59
New onset atrial fibrillation	141 (30.4)	71 (33.6)	70 (27.6)	0.19
Pacemaker implantation	14 (3.0)	7 (3.3)	7 (2.8)	0.79
30-days mortality	23 (4.9)	5 (2.4)	18 (7.1)	0.03
Cardiac-related mortality	11 (2.4)	4 (1.9)	7 (2.8)	0.76
**One year mortality**	47 (10.1)	12 (5.7)	35 (13.8)	0.003
Due to cardiac causes	16 (3.4)	5 (2.4)	11 (4.3)	
Due to non-cardiac causes	18 (3.9)	4 (1.9)	14 (5.5)	
Due to unknown causes	13 (2.8)	3 (1.4)	10 (3.9)	
**Five-year mortality**	84 (18.1)	26 (12.3)	58 (22.9)	0.007
Due to cardiac causes	17 (3.7)	5 (2.4)	12 (4.7)	
Due to non-cardiac causes	36 (7.8)	11 (5.2)	25 (9.9)	
Due to unknown causes	31 (6.7)	10 (4.7)	21 (8.3)	
**Overall mortality**	147 (31.7)	52 (24.6)	95 (37.5)	0.002
Due to cardiac causes	20 (4.3)	5 (2.4)	15 (5.9)	
Due to non-cardiac causes	62 (13.4)	22 (10.4)	40 (15.8)	
Due to unknown causes	65 (14)	25 (11.8)	40 (15.8)	
**Lost during follow-up**	23 (5)	7 (3.3)	16 (13.8)	0.002
**Follow-up time, months**	88.8 ± 39.4	87.3 ± 36.7	89.9 ± 41.4	0.21

Data presented as number (%) or median with interquartile range.

**Table 5:** Clinical follow-up outcomes.

Variable	Total *n* = 294	Isolated–SAVR *n* = 152	Combined-SAVR *n* = 142	*p*-Value
Social history				
Independently patient	216 (73.5)	112 (73.7)	104 (73.2)	0.54
Need help	78 (26.5)	40 (26.3)	38 (26.7)	0.54
Survivals * NYHA classification				
NYHA I-II	220 (74.8)	110 (72.4)	110 (77.5)	0.35
NYHA III-IV	74 (25.2)	42 (27.6)	32 (22.5)	0.35
Stroke	16 (5.4)	9 (5.9)	7 (4.9)	0.8
Myocardial infarction	2 (0.7)	0	2 (1.4)	0.23
* PCI / Stent implantation	6 (2.0)	4 (2.6)	2 (1.4)	0.69
Pacemaker implantation	15 (5.1)	4 (2.6)	11 (7.7)	0.06
New temporary haemodialysis	3 (1.0)	3 (2.0)	0	0.25
Prosthesis dysfunction requiring * Re-SAVR				
Severe prosthesis endocarditis	5 (1.7)	3 (2.0)	2 (1.4)	1.0
Prosthesis dysfunction without * Re-SAVR	12 (4.0)	7 (4.6)	5 (3.5)	0.88
Prosthesis stenosis	3 (1.0)	1 (0.7)	2 (1.4)	
Prosthesis insufficiency	6 (2.0)	4 (2.6)	2 (1.4)	
Prosthesis endocarditis	3 (1.0)	2 (1.3)	1 (0.7)	

Data presented as number (%). * PCI = percutaneous coronary intervention; * Re-SAVR = redo surgical aortic valve replacement; *NYHA = New York Heart Association Classification.

## Supplementary Materials

The following are available online at www.mdpi.com/article/10.3390/jcm10235592/s1, Table S1: Multivariate random survival forest.
