# Supplementary material for: Impact of Non-Valvular Non-Coronary Concomitant Procedures on Outcomes of Surgical Aortic Valve Replacement in Intermediate Risk Patients
Source: J Clin Med. 2021 Nov 28;10(23):5592. doi: 10.3390/jcm10235592 (PMC8658681; doi:10.3390/jcm10235592)
Supplement: Supplementary file 1 [file jcm-10-05592-s001.zip › jcm-1424302-supplementary.pdf]

Journal of
Clinical Medicine

MDPI

**Table S1**: Multivariate random survival forest

| Variable. | Cardiac Mortality *p*-Value | All-Cause Mortality *p*-Value |
|---|---|---|
| Age, years | 0.386 | 0.019 |
| Gender, male | 0.478 | 0.119 |
| BMI*, kg/m$^2$ | 0.668 | 0.648 |
| COPD* | 0.516 | 0.38 |
| Diabetes mellitus | 0.345 | 0.055 |
| Systemic hypertension | 0.328 | 0.201 |
| Pulmonary hypertension | 0.118 | 0.175 |
| Preoperative creatinine | 0.083 | 0.084 |
| Prior myocardial infarction | 0.477 | 0.069 |
| NYHA III-IV | 0.127 | 0.056 |
| Urgent/Emergent indication | 0.218 | 0.002 |
| Logistic EuroSCORE I | 0.802 | 0.701 |
| EuroSCORE II | 0.785 | 0.246 |
| STS-PROM | 0.045 | 0.476 |
| Ejection fraction <50% | 0.143 | 0.035 |
| Cross-clamp time | 0.821 | 0.647 |
| CPB*-time | 0.065 | 0.107 |
| Prothesis size | 0.646 | 0.56 |
| Concomitant SAVR | 0.01 | 0.003 |

*BMI=Body mass index; *COPD=Chronic obstructive pulmonary disease; *NYHA=New York Heart Association, *CPB= Cardiopulmonary bypass.
